# The influence of the cultural climate of the training environment on physicians' self-perception of competence and preparedness for practice

**DOI:** 10.1186/1472-6920-8-51

**Published:** 2008-11-21

**Authors:** Jamiu O Busari, Eduard AA Verhagen, Fred D Muskiet

**Affiliations:** 1 Department of Paediatrics, Atrium Medical Center, Heerlen, Netherlands; 2 Department of Paediatrics, University Medical Center, Groningen, Netherlands; 3 Department of Paediatrics, St. Elisabeth Hospitaal, Breedestraat 193, Curaçao, Netherlands Antilles

## Abstract

**Background:**

In current supervisory practice, the learning environment in which the training of specialist registrars (SpRs) takes place is important. Examples of such learning environments are the hospital settings and/or geographical locations where training occurs. Our objective was to investigate whether the cultural climate of different learning environments influences physicians' perceived level of competence and preparedness for practice.

**Methods:**

An electronic questionnaire was sent to an equal group of paediatricians who had trained in clinical settings located in Europe and the Caribbean. 30 items (Likert scale 1–4 = totally disagree-totally agree) were used to measure the level of preparedness of the respondents in 7 physician competencies.

**Results:**

42 participants were included for analysis. The distribution of participants in both groups was comparable. The overall perception of preparedness in the Caribbean group was 2.93 (SD = 0.47) and 2.86 (SD = 0.72) in the European group. The European group felt less prepared in the competency as manager 1.81 (SD = 1.06) compared to their Caribbean counterparts 2.72 (SD = 0.66). The difference was significant (p = 0.006).

**Conclusion:**

The training in the different environments was perceived as adequate and comparable in effect. The learning environment's cultural climate appeared to influence the physician's perception of their competencies and preparedness for clinical practice.

## Background

The learning environment in which the supervision of specialist registrars (SpRs) takes place is important in current supervisory practice. The learner's perception of the quality of training is also known to be influenced by the environment, in which learning takes place. Studies investigating SpRs professional skills after clinical rotations in different teaching hospitals, showed that certain skills were developed better in specific teaching settings, and non-university teaching hospitals were favoured as effective learning environments than university teaching hospitals [1,2]. In addition, SpRs regarded the learning environment as an important determinant of effective supervision during their clinical rotations [3]. Similar studies have also investigated the impact of learning environment on nursing students' preparedness for practice. Interestingly, those who trained in rural clinical settings felt more competent and confident for their profession than those who trained in metropolitan settings [4]. Although it obvious that the context (physical or conceptual) of learning influences the quality of learning in students, information on how this process occurs and the dynamics involved in the process are still scant.

The training of SpRs occurs in different learning settings e.g. university hospitals or district teaching hospitals, or different environments i.e. geographical locations within or outside a country. By "SpR training", we refer to the training of medical residents (or fellows) as it occurs in the United states and Canada. In the Netherlands, there are 35 hospital settings where the training of paediatric SpRs takes place. The hospital settings are made up of 8 university medical centres (UMC) and 27 district teaching hospitals (DTH). One of these DTH's is located outside the Netherlands. Each DTH is affiliated to a UMC and in order to gain the exposure of practicing outside a predominantly research-driven UMC, SpRs spend between 1 to 2 years of their 5 year residency training in a DTH. It is believed that the experiences residents' acquire in both the university and non-university clinical settings contribute towards an all-round professional development necessary for their future career choice.

### Context of the survey: UMC, Groningen, the Netherlands

Like in many other countries, the professional training of SpRs in the Netherlands is undergoing major reform. The country's Central College of Medical Specialists (CCMS) has recommended the revision of the country's 33 specialist training programs such that the content sufficiently addresses the growing changes in the medical profession, as well as, the changing demands in health care services by the society [5]. Seven professional competencies were recommended, culled from the essential role and key competencies of specialist physicians of the Royal College of Physicians and Surgeons, Canada. These competencies were intended to constitute a new competency-oriented, training program for Dutch SpRs and included the Medical Expert, Communicator, Collaborator, Manager, Health Advocate, Scholar and Professional [6].

In line with the CCMS recommendations, the UMC in Groningen (UMCG) began with an initial assessment of the (current) quality of training in its six affiliated DTHs, that included one in Curaçao, the Netherlands Antilles. This DTH, the St. Elisabeth hospital, is also affiliated to the UMC in Amsterdam [7]. The aim of this assessment was threefold. First, to obtain a baseline impression of the quality of professional training in all the DTH's affiliated to the UMCG before implementing curricula reforms. Secondly, to objectively determine whether all affiliated DTHs satisfied the requirements for serving as teaching hospitals and thirdly, to determine whether the training in the DTH in Curaçao influenced the preparation of its graduates prior to professional practice, as it was the only teaching hospital setting where the training occurred in a different learning environment i.e. in the Caribbean. The following research questions were formulated for this survey:

1. Does the training in all DTH's, prepare SpRs in the seven intended professional competencies?

2. Which of the seven competencies requires improvement in the DTHs?

3. In what way, if any, does learning environment influence the perceived level of preparedness (PLP) of SpRs?

## Methods

### Participants

A total of 89 practicing paediatricians were identified as eligible to participate in this survey, 43 of whom had undergone their non-university clinical rotation in Curaçao. All participants were Dutch medical graduates from either the university medical centre of Groningen or Amsterdam, as the DTH in Curaçao was affiliated to both institutions. The training they underwent was based on the residency training curriculum as defined by the Dutch paediatric society. We selected our partcipants (i.e. graduates as far back as 1985) from the register of the paediatric society of the Netherlands and enlisted them into the survey between July and August of 2005. Written informed consent was sought from the participants prior to the survey and was obtained as such. The study design was set up in compliance with the Dutch national regulations for medical privacy and medical research. Ethical approval was not sought for as this was not applicable for the study.

Since the objective of this survey included identifying any influence the learning environment (i.e. dependent variable) may have had on the respondents' perceptions of professional competency, we divided the selected participants into two groups, i.e. those who trained in a DTH in the Netherlands (DTHN) and those who trained in the DTH in Curaçao (DTHC). Quality of training in this survey was described as how well physicians felt prepared in the seven professional competencies as defined by CCMS/CanMeds (i.e. outcome variable).

In order to obtain an impression of how the training in the different learning environments influenced the perceived level of preparation of the graduates, we chose to conduct a retrospective survey using a standardised questionnaire for this purpose. We chose upfront to accept the potential limitations associated with respondents' recall of prior experiences taking into account the importance of the information we needed.

As we knew beforehand that we had a relatively small pool of graduates in the DTHC group, a "compromise power analysis" was performed to determine the minimum number of participants that would generate reliable findings from the survey. Compromise power analysis represents a novel concept in statistics, which can be determined using the G*Power software package. It is applicable in uncontrollable situations (e.g., working with clinical populations), or when N is too small to satisfy conventional levels of alpha and beta (1-power) given the effect size [8]. As both were the case in our situation, we used this method to determine the number of participants we would need for our survey as well as for the study's statistical power. The minimum number participants needed to achieve a power of 0.90, with a high effect size of 0.8 and β/α ratio = 4 was calculated from the 89 eligible paediatricians. The outcome showed that 40 participants (20 in each group), would yield a statistical power of 0.89 for our survey (Alpha: 0.05, Effect Size: 0.8). We sent questionnaires to each paediatrician via email and were able to recruit 42 participants within the 2 month period of the survey. This was enough to satisfy the minimum number of participants that were required.

### Measurement instrument

We designed a 37 item questionnaire that reflected how the respondents felt their training in the DTH contributed to their overall professional development. 28 of these items specifically reflected the seven core competencies as recommended by the CCMS for the Dutch medical specialists training program. Using a likert scale of 1 = totally disagree and 4 = totally agree, the respondents responded to 4 items that reflected their level of confidence in each desired professional competencies (see additional file 1). The reliability of the questionnaire and internal consistency of these items were tested in a separate pilot study (unpublished) and were shown to be high (Cronbach's alpha = 0.96; average measure intra-class correlation of the items = 0.96 (95% CI 0.92–0.99). The respondents were also given the opportunity to elaborate on their responses in an open section of the questionnaire. All the questionnaires were distributed and returned via email.

### Data analysis

We conducted the analysis of the data using 36 questionnaires. Six of the questionnaires were excluded because 4 respondents rated essential items incorrectly and/or left out important demographic information e.g. year of graduation, training location, that was vital for the analysis. Two other respondents who were excluded turned out to have participated in a different programme other than the mainstream residency training. Post hoc power analysis of the remaining 36 questionnaires was performed and revealed an acceptable power of 0.76 (Alpha: 0.05, Effect Size: 0.8). Descriptive statistics were used to interpret the responses of the respondents. Means and standard deviations were calculated for the perceived quality of the training in the different competency subscales, and also for the overall level of preparedness (i.e total mean of the 28 items of describing the different competencies). Students independent T-test was used to identify differences in the perceived quality of training between the learning environments. Significance was defined as a p-value < 0.05. We used analysis of variance (ANOVA) to examine whether there were differences in the respondents' perceptions based on year of graduation or choice of clinical practice i.e. university or general hospital setting. We investigated for any significant differences across and between graduation year group and type of hospital setting. Chi-Square analysis was used to identify whether the respondents perceptions were influenced by variables such as age, sex, year of graduation, or sub-specialization. Multiple regression analysis was used to identify the competency that was the best predictor of overall professional competency. Statisical analysis was performed using the SPSS 12.01 software package.

## Results

There was a fairly balanced demographic distribution among the participants in this study. Half of the respondents had undergone their non-university clinical training in Curaçao. The distribution of other half of the respondents that trained in the Netherlands was spread over 3 different teaching hospitals in a group of 4, 5, and 9 respectively. The majority of the respondents were employed in teaching hospital settings, with the largest group in university teaching hospitals (n = 15, 42%). Most of them (n = 15, 42%) were in the age group of 40–49 years and graduated in the period 1990–1999 (n = 15, 43%). (See table 1).

**Table 1:** Demographic distribution of all respondents

**Profile**	**Subgroup**	**Frequency**	**%**
Age group	30–39	10	28
	40–49	15	42
	50–59	11	31
Sex	Male	22	61
	Female	14	39
Graduation period (year)	1980+	11	31
	1990+	15	42
	2000+	10	28
Sub-specialist	Yes	22	61
	No	14	39
Current employment	University teaching hospital	15	42
	District teaching hospital	12	33
	General hospital	9	25
District Teaching Hospital setting	Netherlands	18	50
	Netherlands Antilles	18	50

### Influence of the training in the district teaching hospitals on level of preparedness

In general, the paediatricians' felt that the training in the DTH's contributed reasonably to their overall level of preparedness (overall mean: 2.90; SD: 0.60). They agreed that the training in the DTH was a valuable experience (mean 3.74; SD 0.57). The competencies that were rated highly were "professional" (mean 3.05; SD 0.62) and "communicator" (mean 3.05; SD 0.63), while "scholar" was the competency rated lowest (mean 2.63, SD: 0.90). The best predictor of overall competency was medical expert (F = 108.7, P = 0.00) and accounted for 78% of the total variance (R^2 ^= 0.78). The item "being able to offer effective and ethically responsible patient care" was rated highest in the questionnaire (mean 3.21; SD 0.65) while "working effectively and purposefully within the health care organization" was rated lowest (mean 1.75; SD 1.51).

The respondents felt that that the training in the DTH was responsible for as much as 50% of their total professional training (mean 3.73; SD 0.58). When asked to rank the different competencies on a scale of 1 to 7 in ascending order of importance (1 = least, 7 = most), the respondents felt that the competency their training prepared them best in was as "medical expert" (mode 7, Median 7) and least as "manager" (mode 1, median 2.5)

Chi-square analysis showed that none of the demographic characteristics (year of graduation, age group, gender, professional training, subspecialty) influenced the respondents' ratings. Analysis of variance failed to show any significant differences between groups based on year of graduation, age and training setting. However, the significance of these findings should be interpreted with caution, considering the relatively small size of the participants.

### Influence of learning environment on perceived level of preparedness

The paediatricians who trained in Curaçao (DTHC) felt well prepared for clinical practice through their training and in the seven different competencies as physicians. Their colleagues who underwent the training in the Netherlands (DTHN) however, felt less prepared in two of the seven competencies, namely, as scholar (2.35, SD: 1.02) and manager (mean 1.81, SD: 1.06). The difference between the respondents PLP as manager was significant (t = -2.96, df = 31, p = 0.006) when compared to their counterparts who trained in Curaçao. There was no significant difference between the DTHC (mean 2.93, SD 0.43) and DTHN (mean 2.86, SD 0.72) groups with respect to how much the training in district teaching hospitals contributed to their professional development (1 = totally disagree, 4 = totally agree). The competency that scored highest in the DTHC group was "Health advocate" (mean 3.00, SD 0.50) while the competency as "manager" scored lowest (mean 2.72 SD 0.66). The competency that was rated highest within the DTHN group was "communicator" (mean 3.25, SD 0.68) while the competency that was rated lowest was "manager" (mean 1.81, SD 1.06) (see table 2).

**Table 2:** Comparison of the respondents' perceptions per location on level of preparedness in the different competencies

**Competency**	**District Teaching Hospital Setting**	**N**	**Mean**	**SD**	**Range**	**95% CI**
Medical Expert^†^	Netherlands	16	2.84	0.72	2.50	2.46–3.23
	Curaçao	17	2.78	0.55	2.25	2.50–3.06
Communicator	Netherlands	16	3.25	0.68	2.00	2.89–3.61
	Curaçao	17	2.87	0.52	1.75	2.60–3.13
Collaborator	Netherlands	16	2.86	0.64	2.25	2.52–3.20
	Curaçao	17	2.75	0.56	2.00	2.46–3.03
Scholar	Netherlands	16	2.36	1.02	4.00	1.81–2.90
	Curaçao	17	2.88	0.70	2.50	2.52–3.24
Health Advocate	Netherlands	16	2.81	0.83	2.50	2.37–3.25
	Curaçao	17	3.00	0.50	2.00	2.74–3.26
Manager*	Netherlands	16	1.81	1.06	3.75	1.25–2.38
	Curaçao	17	2.72	0.66	2.25	2.38–3.06
Professional	Netherlands	16	3.12	0.74	2.25	2.73–3.52
	Curaçao	17	2.99	0.52	2.00	2.72–3.25
**Level of Preparedness**	Netherlands	16	2.86	0.72	2.40	2.43–3.15
	Curaçao	17	2.93	0.47	1.76	2.69–3.17

Scale: (1 = totally disagree, 2 = disagree, 3 = agree, 4 = totally agree)

* = The difference between the means was significant (p = 0.006)

^† ^= Best predictors of level of preparedness using multiple linear regression (R^2 ^= 0.78)

Asked if they considered their non-academic postings valuable (1 = totally disagree, 4 = totally agree), both the DTHN (mean 3.75, SD 1.12) and the DTHC respondents agreed that the posting was valuable for their professional development (mean 4.12, SD 1.17) (see table 3). Finally, when asked to rank the competency they felt they were best prepared in through their non-academic residency postings in ascending order of importance (1 = least, 7 = most), the DTHC respondents ranked Medical Expert (mode 7, median 7) and Collaborator (mode 6 median 5) as highest while the DTHN group ranked Medical Expert (mode 7, median 7) and Scholar (mode 6, median 5) as highest.

**Table 3:** Respondents' perceptions of the value of the rotation to their professional development.

**Comment**	**District Teaching Hospital Setting**	**N**	**Mean**	**SD**
I found the clinical rotation valuable^#^	Netherlands	16	3.65	0.61
	Curaçao	17	3.82	0.53
The contribution of the training in the district teaching hospital to my overall professional development was*	Netherlands	16	3.75	1.13
	Curaçao	17	4.12	1.17

^# ^Scale: (1 = totally disagree, 2 = disagree, 3 = agree, 4 = totally agree)

* Scale: (1 = < 30%, 2 = 30–40%, 3 = 40–50%, 4 = 50–60%, 5 = > 70%)

### General comments on the contribution of the residency-location to perceived level of preparedness for practice

The general comments that were provided by reflected that respondents perceived that the training in the district teaching hospitals contributed to their professional development in the competencies as medical expert, manager and collaborator (see table 4).

**Table 4:** General comments reflecting respondents perception of the contribution of residency-location to their level of preparedness for practice

Respondents	Comments
DTH Curaçao	"I learnt a lot from my supervisors, I was stimulated to study a lot and my knowledge was constantly prodded during rounds....." "I developed more competencies during my posting in the district teaching hospital, compared to my rotations in the university teaching hospital. I also learnt (more) professionally relevant skills in the district teaching hospital"
	"This was a good professional training experience that enabled me to develop extensive clinical skills for in- and out-patient care, adapt to a different culture, as well as, to the delivery of health care in a different context. It enabled me to enhance my problem solving abilities, my creativity and also learn how to cope with my frustrations and responsibilities"
	
	"What was important for me was the social context here (Curaçao) that you had to reckon with. The different culture enables you to understand that Dutch norms and values do not apply to everyone, and this should be borne in mind.....you learn to work independently and manage your patients alone without the interference of sub-specialists"
	
DTH Netherlands	"I found the rotation very valuable..... I was able to apply the acquired theoretical knowledge in practice, as well as, integrate knowledge, skills and attitudes into a whole unit.... You learn how to become a doctor in the district hospital setting, you are able to identify and define your limitations, professional independence, self education, etc."
	
	"Although it was a long time ago, what I still recollect is that it was an extremely valuable training... during the period of the training, my sense of professional autonomy and accountability grew enormously"

## Discussion

This survey describes physicians PLP for practice and their competence in 7 physician roles following their professional training in district teaching hospitals. It also investigates whether noticeable differences are present that could be attributed to the different learning environments. In general, the respondents perceived that the professional training in the district teaching hospitals prepared them well in the 7 physician competencies as described in CanMeds 2000. As much as 50% of their total professional development during the residency was attributed to the training in the DTHs. Although, certain competencies were perceived to be better developed in the different settings by the respondents, the overall perception of competence and preparedness for practice was comparable in both groups. The professional competency that the respondents felt best prepared in, was as medical expert. It was also found to be a good predictor of overall level of competency.

There were differences observed in the PLP between the two groups. The DTHN respondents felt less prepared as scholars and managers compared to the DTHC who felt well prepared in all of the professional competencies. Looking at the competencies they felt prepared in, the DTHN respondents felt better prepared in their roles as medical expert, communicator and professional, while their counterparts who trained in Curaçao felt better prepared in the competencies as manager and health advocate. Overall, the DTHC group felt slightly well prepared than their DTHN counterparts. They experienced that the training in the DTH contributed slightly more to their professional development than the DTHN respondents.

The number of studies in the literature on the influence of learning environment on professional development is scant. However, it has been shown for example, that training in rural settings stimulates greater competence, confidence and organisational skills in nursing students compared to those who train in the Metropolitan setting [4]. The findings in the survey we have conducted reflect that there are differences in the perceived level of competence and preparedness between the two groups. Unfortunately, it is difficult to offer an unequivocal explanation for the differences based on these findings. Nonetheless, it is our assumption that certain tacit factors may have had an influence on our results.

It is known that the organizational climate and culture of communities tend to influence the way different communities coordinate their activities [7]. While the more general dimensions of national health systems in different countries reflect universal models and change in similar directions, there are clear national differences in specific health care structures and policies, particularly in those concerning the availability and utilization of national health care resources [9,10]. Nonetheless, the choices of many national and local interest groups in defining their health care structures are influenced by processes similar to those described by Hofstede i.e. power-distance, uncertainty-avoidance, individualism and masculinity [7].

Judging by some of the open comments our respondents provided, some of the observed differences in this study could be attributed to the environment (i.e. cultural climate) in which the clinical trainings took place. In Curaçao for example, the health care system is less well-structured, the culture of the doctor-patient relationship is paternalistic, and autonomy rests more with the care providers than with the patients themselves. In the Netherlands on the other hand, the health care system is highly specialized and the paternalistic doctor-patient relationship is less evident. In this setting, patients participate more actively in clinical decision-making and more autonomy rests with the patient than with the doctor.

Using the concepts mentioned above, the culture in Curaçao could be characterized as having a large power-distance (i.e. extent to which the community encourages superiors to exert power), masculinity (i.e. extent to which the culture places emphasis on performance and difference in the sexes, than to quality of life) and low individualism (i.e. emotional dependence of individual on organization or institution) [11]. This may explain why patient autonomy is low and why the responsibility for self is placed in the hands of health care providers in such communities. Compared to western societies, health care providers in settings such as Curaçao are obliged to develop good managerial and advocacy skills, in order to assure that the patients obtain optimal health care services. It is our assumption that this process may have facilitated the preferential development of different competencies observed between the two groups of respondents studied in this survey. An alternative hypothesis could also be that the training in managerial skills is poor to begin with in DTHs located in the Netherlands. Unfortunately, we could neither refute nor confirm this claim based on the findings from our survey

A methodological limitation we faced in this survey was the restricted pool of potential participants we could approach (N = 89). Of this group, forty-three had undergone their training in Curaçao since its inception in 1985. The remainder of this batch (N = 46), underwent their residency in different DTHs in the Netherlands. As, the sizes of the different groups in the DTHN group were uneven and small, we could not perform reliable subgroup analysis. Although we considered the use of electronic questionnaires to be an efficient and cost effective method in reaching our target group, the response from the participants was poor, raising the question about the appropriateness of this method.

A point worth clarifying is the choice of the statisitcal test used for the analysis of our results. While one might argue that non-parametric tests would have been a preferred method to use, we chose to use parametric tests instead (t-test and ANOVA). Our choice for this was based on two considerations. Firstly, the frequency distribution of the participants in our survey showed a fairly equal spread in the different demographic categories suggesting a Gaussian distribution. Secondly, it is known that variables scored on likert-scales lie in between ordinal and interval variables, as reflected by the four-point scale we used in our questionnaire i.e. "totally agree", "agree", "disagree" and "totally disagree". In situations where it is uncertain that the intervals between each of these four values are the same, the variables cannot be classified as interval, but rather as ordinal variables. We dealt with this problem by assuming that the interval of our scale was equally spaced. For the sake of completeness, we also performed a Mann Whitney test on our data. The result of the analysis was similar to that of the parametric tests we conducted, with the only significant difference found in the respondents perceived preparedness as manager (66,000; P = 0.01) and which was in favour of the physicians who trained in Curaçao.

Finally, the strength of this survey was in the use of an instrument that was tested to be reliable and the comparable size and the breadth of the post-training period of the respondents in both groups.

## Conclusion

Although we acknowledge the potential weaknesses of our survey, we believe it is one that has made an effort to investigate the influence of learning environment on graduates PLP. Exposed to similar treatment conditions, the results of this survey demonstrate that the training in the different DTHs was adequate and comparable in effect. Certain competencies were perceived to be better developed in particular learning environments. Furthermore, it was shown that the learning environment influenced physicians' PLP in different competencies and for clinical practice. We acknowledge that it is difficult to explain the extent of the influence of the learning environment on the physicians' PLP; however, the results do provide an understanding of the cultural dynamics of the educational environment on higher medical education. We believe the experiences and findings from this survey can be of support to other investigators with similar research questions.

## Competing interests

The authors declare that they have no competing interests.

## Authors' contributions

JOB conceived and designed the study, made a substantial contribution to the writing of the article, analysis and interpretation of the data, and revision of the article critically for important intellectual content. AEV made substantial contributions to the conception and design of the study, acquisition of data, and revision of the article critically for important intellectual content. FDM made substantial contributions to the conception and design of the study, and revision of the article critically for important intellectual content. All authors read and approved the final manuscript.

## Pre-publication history

The pre-publication history for this paper can be accessed here:



## Supplementary Material

Additional file 1 **Appendix 1.** Items used to assess physicians' perceptions of their preparedness in the different competencies. Click here for file
