# Supplementary material for: The influence of the cultural climate of the training environment on physicians' self-perception of competence and preparedness for practice
Source: BMC Med Educ. 2008 Nov 21;8:51. doi: 10.1186/1472-6920-8-51 (PMC2596784; doi:10.1186/1472-6920-8-51)
Supplement: Additional file 1 — Appendix 1. Items used to assess physicians' perceptions of their preparedness in the different competencies. [file 1472-6920-8-51-S1.doc]

Appendix 1.

**Items used to assess physicians’ perceptions of their preparedness in the different competencies**

| (Scale: 0=not applicable; 1=totally disagree; 2=disagree; 3=agree; 4=totally agree |
| --- |
| **Medical Expert** |
| 1. Appropriately apply the profession’s diagnostic, therapeutic and preventive arsenal and where possible, based on available evidence 2. Provide effective and ethically responsible health care service 3. Possess adequate knowledge and skills relevant for the specialty 4. Promptly find and appropriately apply the required medical information for optimal patient management |
| **Communicator** |
| 1. Adequately discuss relevant medical information with the patient and if necessary with relatives 2. Obtain relevant and purposeful information from patients/guardians through good listening skills 3. Build effective professional relationships with patients 4. Adequately prepare verbal and written reports on patient cases |
| **Collaborator** |
| 1. Contribute to an effective interdisciplinary cooperation and health care network service 2. Adequately refer patients to other health care givers 3. Purposefully communicate with colleagues and other health care providers 4. Effectively handle inter-collegial consultations |
| **Scholar** |
| 1. Develop and maintain my personal continuing medical education 2. Promote the professional abilities of students, colleagues, and other partners in the health care system 3. Adequately develop and expand my scientific/professional knowledge 4. Critically appraise medical information |
| **Health Advocate** |
| 1. Perform my duties in line with the relevant (local) judicial guidelines 2. Promote the general well being of the patient and community as a whole 3. Adequately respond to health care related incidents 4. Recognize and identify the determinants of illness and disease |
| **Manager** |
| 1. Responsibly utilize the available resources for patient management 2. Work effectively and intentionally within the health care organization 3. Organize my work to achieve a balance between patient care and personal development 4. Use information technology for optimal patient care as well as for my own continuing professional training |
| **Professional** |
| 1. Exhibit adequate personal and interpersonal professional conduct 2. Identify the limitations of my own professional competency and conduct 3. Practice medicine in line with the ethical norms customary to my profession 4. Deliver health care services reliably, truthfully and empathetically |
| **General** |
| 1. I found the training in the District Teaching Hospital very valuable   (Scale: 0=not applicable; 1=totally disagree; 2=disagree; 3=agree; 4=totally agree) |
| 1. The contribution of the training in the District Teaching hospital to my overall professional development was   (Scale: 1 =< 30%; 2 =30-40%; 3 =40-50%; 4 =50-60%; 5 =>70%) |
